# Prioritizing CircRNA–Disease Associations With Convolutional Neural Network Based on Multiple Similarity Feature Fusion

**DOI:** 10.3389/fgene.2020.540751

**Published:** 2020-09-16

**Authors:** Chunyan Fan, Xiujuan Lei, Yi Pan

**Affiliations:** ^1^School of Computer Science, Shaanxi Normal University, Xi’an, China; ^2^Department of Computer Science, Georgia State University, Atlanta, GA, United States

**Keywords:** circRNA-disease associations, circRNA-miRNA interaction, similarity kernel fusion, feature matrix, convolutional neural network

## Abstract

Accumulating evidence shows that circular RNAs (circRNAs) have significant roles in human health and in the occurrence and development of diseases. Biological researchers have identified disease-related circRNAs that could be considered as potential biomarkers for clinical diagnosis, prognosis, and treatment. However, identification of circRNA–disease associations using traditional biological experiments is still expensive and time-consuming. In this study, we propose a novel method named MSFCNN for the task of circRNA–disease association prediction, involving two-layer convolutional neural networks on a feature matrix that fuses multiple similarity kernels and interaction features among circRNAs, miRNAs, and diseases. First, four circRNA similarity kernels and seven disease similarity kernels are constructed based on the biological or topological properties of circRNAs and diseases. Subsequently, the similarity kernel fusion method is used to integrate the similarity kernels into one circRNA similarity kernel and one disease similarity kernel, respectively. Then, a feature matrix for each circRNA–disease pair is constructed by integrating the fused circRNA similarity kernel and fused disease similarity kernel with interactions and features among circRNAs, miRNAs, and diseases. The features of circRNA–miRNA and disease–miRNA interactions are selected using principal component analysis. Finally, taking the constructed feature matrix as an input, we used two-layer convolutional neural networks to predict circRNA–disease association labels and mine potential novel associations. Five-fold cross validation shows that our proposed model outperforms conventional machine learning methods, including support vector machine, random forest, and multilayer perception approaches. Furthermore, case studies of predicted circRNAs for specific diseases and the top predicted circRNA–disease associations are analyzed. The results show that the MSFCNN model could be an effective tool for mining potential circRNA–disease associations.

## Introduction

Circular RNAs (circRNAs) are a type of endogenous non-coding RNA with continuous covalently closed loop structures, which are produced by back-splicing or lariat events in genes (Barrett et al., 2015). Recently, with the development of high-throughput sequencing techniques and other technologies, a large number of circRNAs have been found in various organisms, including protists, plants, and metazoans (Danan et al., 2012; Memczak et al., 2013; Tang et al., 2018). The main functions of circRNAs include sequestration of microRNAs (miRNAs) and proteins (Salmena et al., 2011), regulation of transcription and splicing (Zhang et al., 2013; Conn et al., 2017), and even translation to produce polypeptides (Yang et al., 2017; Sun and Li, 2019). Accumulating evidence implicates mutation or alteration in expression of circRNAs in the initiation and progression of numerous diseases. For example, Chioccarelli et al. (2019) identified the differentially expressed circRNAs in human spermatozoa, and found that circRNAs are related to spermatozoa quality. By comparing the expression profiles of circRNAs in disease-specific tissues or cell lines with those in normal samples, significantly increased or decreased circRNAs can be identified. In addition, the intrinsic characteristics of circRNAs indicate they are stable both inside cells and in extracellular plasma (Bahn et al., 2015; Li et al., 2015; Memczak et al., 2015). Therefore, disease-associated circRNAs are considered to be promising novel biomarkers for diseases.

Recently, several studies have analyzed the roles of circRNAs in varies samples, and further explore their diversity, expression patterns, co-expression network, and so on. circAtlas integrates the most comprehensive circRNAs, their expression, and functional profiles in vertebrates (Wu et al., 2020). MiOncoCirc is a cancer-focused circRNA resource to be generated from an extensive array of tumor tissues (Vo et al., 2019). Ji et al. (2019) identifies full-length transcripts and evolutionarily conserved circRNAs, and infers circRNA functions on a global scale. Ruan et al. (2019) characterizes circRNAs expression profiles, and explores the potential mechanism of circRNA biogenesis as well as its therapeutic implications. exoRBase integrates and visualize the RNA expression profiles both normal individuals and patients with different diseases (Li et al., 2018). These studies will trigger functional implication for human diseases and benefit biomedical research community.

The de-regulated circRNAs in diseases can be identified for validation using low-throughput biological methods such as quantitative real-time PCR, northern blotting, and so on. However, these traditional experiments are costly and time-consuming. Therefore, computational approaches are important for exploring potential disease-causing circRNAs and understanding the associated mechanisms of pathogenicity. Several models have been proposed to forecast circRNA–disease associations; most of these approaches are based on the assumption that circRNAs with similar functions are likely to be associated with the same or similar diseases. Lei et al. (2018) developed a path-weighted model to predict circRNA–disease associations based on circRNA semantic similarity and disease functional similarity (Lei et al., 2018). KATZHCDA was used to calculate the number of walks between nodes and walk lengths for circRNA–disease associations, based on *a priori* knowledge of the circRNA expression similarity and disease phenotype similarity (Fan et al., 2018b). DWNN-RLS predicted circRNA–disease associations using regularized least squares of the Kronecker product kernel (Yan et al., 2018). Xiao et al. (2019) proposed a weighted dual-manifold regularized low-rank approximation model for disease-related circRNA prediction, called MRLDC (Xiao et al., 2019). Another model, iCircDA-MF, incorporated circRNA–gene, gene–disease, and circRNA–disease associations, together with disease semantic information, and used non-negative matrix factorization to predict circRNA–disease associations (Wei and Liu, 2019). Zhao et al. (2019) integrated the bipartite network projection algorithm and KATZ measure algorithm to explore novel circRNA–disease associations (Zhao et al., 2019). Deng et al. (2019) combined circRNAs, proteins, and diseases to predict circRNA–disease associations using the KATZ algorithm (Deng et al., 2019). Ge et al. (2019) developed the LLCDC model for prediction of human disease-associated circRNAs using locality-constrained linear coding and a label propagation algorithm (Ge et al., 2019). CD-LNLP calculated circRNA similarity and disease similarity using linear neighborhood similarity based on known associations, and then used the label propagation algorithm to mine circRNA–disease associations (Zhang et al., 2019). Wang Y. et al. (2019) used a graph-based recommendation algorithm, PersonalRank, to predict disease-related circRNAs based on circRNA expression profiles and functional similarities (Wang Y. et al., 2019). Lei and Fang (2019) used a gradient boosting decision tree with multiple biological data fusion for circRNA–disease prediction (Lei and Fang, 2019). Ding et al. (2020) developed the RWLR model based on the random walk and the logistic regression to predict circRNA-disease associations. iCDA-CGR quantified the sequence nonlinear relationship of circRNA by chaos game representation technology based on the biological sequence position information (Zheng et al., 2020). Lei and Bian (2020) integrated the random walk with restart and *k*-nearest neighbors to predict the associations between circRNAs and diseases. Although these computational models have achieved encouraging results, they represent the tip of the iceberg with respect to predicting circRNA–disease associations.

Several circRNAs can bind with the corresponding miRNAs and participate in multiple biological processes synchronously (Qu et al., 2018). Based on this theory, Fang and Lei (2019) used an improved random walk algorithm to predict circRNA–miRNA associations, named KRWRMC (Fang and Lei, 2019). As miRNAs have been implicated in various diseases, we consider that miRNA information should be included in the identification of circRNA–disease associations. However, there have been few studies of circRNA–miRNA interactions, and deep interaction patterns are rarely considered in prediction of circRNA–disease associations. In this work, we take circRNA–miRNA interactions and miRNA–disease associations into account, and capture the complex miRNA-based interaction features of circRNAs and diseases, respectively.

In recent years, deep learning architectures have attracted increasing attention in various fields, including image analysis (Yang and Xu, 2020), speech recognition (Graves et al., 2013), and bioinformatics (Min et al., 2017), etc. The convolutional neural network (CNN) is a well-known feed-forward artificial neural network inspired by biological processes that simulates the cognition function of human neural systems (LeCun et al., 2015). CNN architectures have the ability to automatically learn the meaning of combinations of features from the input data and simplify the process of manual feature selection (Liu et al., 2017). Recent applications of CNN-based methods indicate their effectiveness in computational biology (Liu et al., 2018), including in circRNA research. Wang and Wang (2019) developed the DeepCirCode model to discover the sequence code of back-splicing for circRNA formation, and sequence motifs were also extracted. The CSCRSites model was proposed to predict cancer-specific protein binding sites on circRNAs based on CNNs. The features learned by the CSCRSites model are converted to sequence motifs, some of which are involved in human diseases (Wang Z. et al., 2019). Inspired by the superior prediction performance of this approach, we used CNN architecture to detect combinations of features and predict potential circRNA–disease associations.

In this study, we present a novel computational model to predict potential associations between circRNAs and diseases, named MSFCNN. The main attributes of the MSFCNN model are as follows. (1) Four circRNA similarity kernels and seven disease similarity kernels are constructed using multiple biological and topological information, such as circRNA expression profiles, circRNA sequence information, disease-miRNA interactions, etc. (2) Whereas some existing methods simply use linear weighting to integrate the similarity kernels into one kernel, we considered that this may lead to information loss and noise. Hence, we used the similarity kernel fusion (SKF) method to fuse four circRNA similarity kernels and seven disease similarity kernels, thereby retaining the original information of each similarity kernel. A weight matrix is used to reduce the noise in the fused similarity kernel. (3) A feature matrix is constructed based on the fused circRNA similarity kernel, fused disease similarity kernel, and interactions and features among circRNAs, miRNAs, and diseases. Multiple biological premises are used to construct the feature matrix. On the one hand, two circRNAs (or diseases) are more similar could capture the relationships between the circRNA (or disease) similarities and circRNA–disease associations. On the other hand, circRNA–miRNA and miRNA–disease associations are also integrated, and the interaction features are captured using principal component analysis. (4) A two-layer CNN architecture is used to process the feature matrix and predict potential circRNA–disease associations. Five-fold cross-validation (CV) is used to assess the prediction performance of the MSFCNN model. The results indicate that the MSFCNN model outperforms several conventional machine learning classifiers. Furthermore, case studies of breast cancer, colorectal cancer, hepatocellular carcinoma, and acute myeloid leukemia indicate that MSFCNN could be an effective tool to infer potential circRNA–disease associations.

## Materials and Methods

**FIGURE 1:**
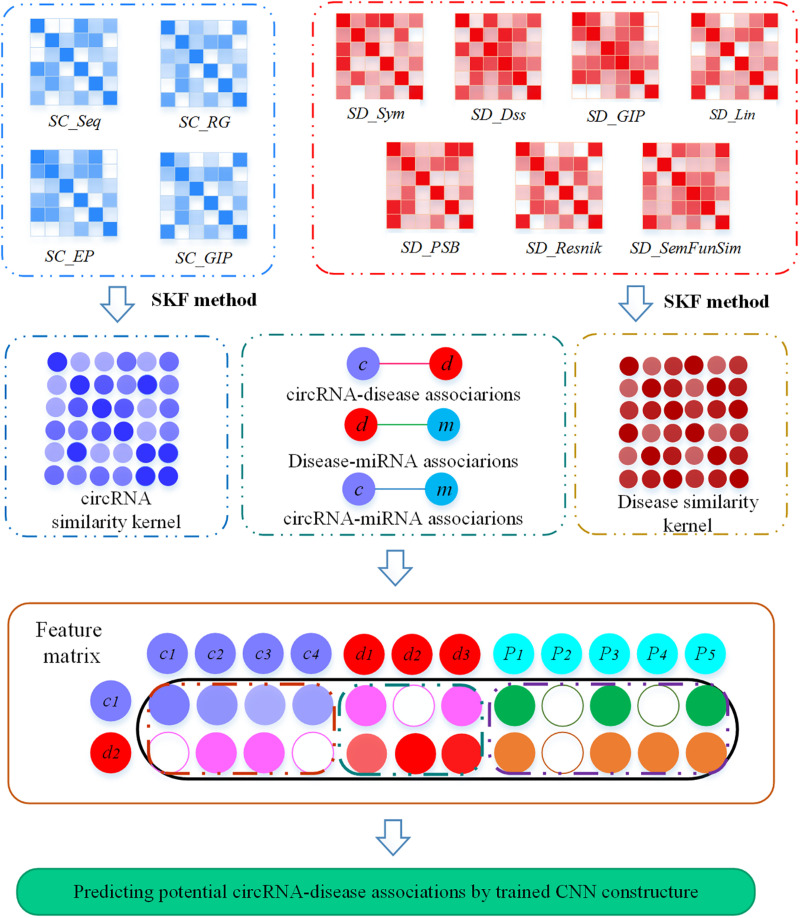
Flow chart of the MSFCNN approach. Step 1: Four circRNA similarity kernels and seven disease similarity kernels are measured, respectively. Step 2: The similarity kernels for circRNAs (or diseases) is fused with SKF method. Step 3: The feature matrix for each circRNA–disease pair is constructed by integrating the fused similarity kernels, interactions and features among circRNAs, miRNAs, and diseases. Step 4: a CNN architecture is used to train MSFCNN approach and predict latent circRNA–disease associations.

A flow chart illustrating MSFCNN, our novel approach to predict potential circRNA–disease associations is shown in Figure 1. First, four circRNA similarity kernels and seven disease similarity kernels are computed based on their biological and topological properties. Then, these kernel similarities are combined into one circRNA similarity kernel and one disease similarity kernel by applying a similarity kernel fusion strategy. Subsequently, the feature matrix can be constructed based on the fused similarity kernels, and interactions and features among circRNAs, miRNAs, and diseases. Finally, we use a CNN to process the feature matrix and predict final scores for prediction of potential circRNA–disease associations.

### Construction of the CircRNA–Disease, CircRNA–miRNA, and Disease–miRNA Networks

In this study, circRNA–disease associations, circRNA–miRNA associations, and disease–miRNA associations were used to predict circRNA–disease associations. Known circRNA–disease associations were downloaded from the CircR2Disease database (Fan et al., 2018a), which contained 739 entries including 725 experimentally validated circRNA–disease associations from four species. Only human circRNA–disease associations were used in this work. Interactions that did not correspond to circRNAs IDs in the circBase database and disease names were not recorded in the disease ontology database were removed (Glazar et al., 2014; Schriml et al., 2019). Thus, we retained 325 circRNAs, 53 diseases, and 371 circRNA–disease associations as the positive dataset. The circRNA–miRNA interactions were obtained from the CircBank database (Liu et al., 2019), and interactions overlapping with disease-related circRNAs were extracted. Thus, 24745 interactions between 322 circRNAs and 2545 miRNAs were obtained. In addition, the disease–miRNA associations that matched circRNA-related diseases were selected from the human microRNA disease database (Huang et al., 2019), and 4970 associations between 37 diseases and 873 miRNAs were obtained. Finally, all of these associations contained three types of nodes including 325 circRNAs, 53 diseases, and 3175 miRNAs.

Based on the circRNA–disease associations, an adjacency matrix *A*(*i*,*j*) was constructed to represent associations between *n*_*c*_ circRNAs and *n*_*d*_ diseases; *A*(*i*,*j*) was assigned a value of 1 if circRNA *c*(*i*) was found to be related to disease *d*(*j*), and 0 otherwise. Similarly, a circRNA–miRNA matrix *Y*(*i*, *j*) was constructed to represent the associations between *n*_*c*_ circRNAs and *n*_*m*_ miRNAs, and the associations between *n*_*d*_ diseases and *n*_*m*_ miRNAs were represented by matrix *O*(*i*, *j*). *Y*(*i*, *j*) was set to 1 when there was an association between circRNA *c*(*i*) and miRNA *m*(*j*), and 0 otherwise. If disease *d*(*i*) interacted with miRNA *m*(*j*), *O*(*i*, *j*) was set to 1, otherwise it was set to 0.

### Representation of CircRNA Similarity Kernels

#### CircRNA Sequence Similarity

The 325 circRNA sequences were obtained from the circBase database (Glazar et al., 2014), and the sequence similarity of each circRNA–circRNA pair was calculated using a modification of the Needleman–Wunsch algorithm with the Emboss-stretcher tool (Rice et al., 2000). Therefore, the circRNA sequence similarity score *SC_Seq*(*c*_*i*_, *c*_*j*_) could be obtained by setting the parameters as follows: Matrix = EDNAFULL, Gap open = 16, Gap extend = 4.

#### CircRNA Regulatory Similarity

Based on the assumption that circRNAs associated with the same miRNAs tend to have similar biological regulatory functions, we used the miRNA–circRNA interactions to measure the circRNA regulatory similarity (Huang et al., 2018). Given the two sets of miRNAs, *M*_*i*_ and *M*_*j*_, that had relationships with circRNAs c*_*i*_* and c*_*j*_*, respectively, the circRNA regulatory similarity kernel was calculated as follows:

(1) $$SC\_RG\left(c_{i},c_{j}\right)=\frac{card\left(M_{i}⋂M_{j}\right)}{\sqrt{card\left(M_{i}\right)}\cdot \sqrt{card\left(M_{j}\right)}}$$

#### CircRNA Expression Similarity

The circRNA expression profiles were derived from the exoRBase database (Li et al., 2018). Each circRNA record had 90 dimensions, representing the expression levels of a single type of circRNA. By extracting the common circRNAs between the CircR2Disease and exoRBase databases, circRNA expression profiles were obtained for calculation of the circRNA similarity kernel. We used the Pearson correlation coefficient to measure circRNA expression similarity, and let *SC_EP*(*c*_*i*_, *c*_*j*_) represent the expression similarity score between circRNAs *c*_*i*_ and *c*_*i*_. The expression similarity kernel of the circRNAs was computed as follows:

(2) $$SC\_EP\left(c_{i},c_{j}\right)=\frac{\sum_{i=1}^{N}\left(xi-\bar{x}\right)\left(yi-\bar{y}\right)}{\sqrt{\sum_{i=1}^{N}{\left(xi-\bar{x}\right)}^{2}\sum_{i=1}^{N}{\left(yi-\bar{y}\right)}^{2}}}$$

where *N* represents the number of properties of the expression profiles, and x*_*i*_* and *y*_*i*_ denote the expression values in different tissues. In general, a pair of circRNAs with a higher correlation score are considered to be more similarly expressed.

#### GIP Kernel Similarity for CircRNAs

The Gaussian interaction profile (GIP) kernel similarity was used to measure the similarity between circRNAs, based on the assumption that similar circRNAs are more likely exhibit a similar interaction or non-interaction pattern with miRNAs (Van Laarhoven et al., 2011). GIP kernel similarity for circRNAs was measured based on circRNA–miRNA interactions and defined as:

(3) $$\begin{array}{c} SC\_GIP\left(c_{i},c_{j}\right)=exp\left(-\gamma_{c}{∥c\left(i\right)-c\left(j\right)∥}^{2}\right)\\ \gamma_{c}=\frac{1}{\frac{1}{n_{c}}\sum_{i=1}^{n_{c}}{∥c\left(i\right)∥}^{2}} \end{array}$$

where the circRNA interaction profiles are represented by *c*(*i*), a binary vector that encodes the interaction between circRNA *i* and all miRNAs, i.e., the *i*-th row of the circRNA–miRNA interaction matrix *Y*. The parameter *γ_*c*_* controls the kernel bandwidth, and *n*_*c*_ is the number of circRNAs.

### Representation of Disease Similarity Kernels

#### Disease Symptom Similarity

According to the co-occurrence of disease and symptom terms recorded in the PubMed bibliography, Zhou et al. (2014) considered that diseases are connected if they have a positive symptom similarity (Zhou et al., 2014). Thus, the disease similarity could be measured and a symptom-based human disease network was constructed. Here, the symptom-based disease similarity *SD_Sym* was obtained from the symptom profiles of diseases.

#### Disease Semantic Similarity

According to Medical Subject Headings descriptions, diseases can be described by a hierarchical directed acyclic graph (DAG). Here, disease semantic similarity is calculated using the method of Wang et al. (2007). DAG*_*d*_* = (*d*, *T*_*d*_, *E*_*d*_) represents the DAG of a disease, in which *T*_*d*_ denotes node *d* and its ancestor nodes, and *E*_*d*_ denotes the direct edges from a parent node to child nodes within *T*_*d*_. Therefore, the semantic contribution of parent node *t* to *d* is defined as follows:

(4) $$D_{d}\left(t\right)=\left\{ \begin{array}{cc} 1, & \text{if}t=d\\ \max\{\Delta *D_{d}\left(d^{\prime }\right)|d^{\prime }\in \mathrm{children}\mathrm{of}t, & \text{if}t\neq d \end{array}\right.$$

where △ represents the semantic contribution decay factor (△ is set as 0.5). The semantic value of disease *d* can be calculated as follows:

(5) $$DV\left(d\right)=\sum_{t\in T_{d}}D_{d}\left(t\right)$$

If two diseases share a larger part of DAGs, they tend to have higher similarity. The similarity score between *d*_*i*_ and *d*_*j*_ is defined as:

(6) $$SD\_Dss\left(d_{i},d_{j}\right)=\frac{\sum_{t\in Td_{i}⋂Td_{j}}\left(D_{d_{i}}\left(t\right)+D_{d_{j}}\left(t\right)\right)}{DV\left(d_{i}\right)+DV\left(d_{j}\right)}$$

#### GIP Kernel Similarity for Diseases

Similar to the calculation of GIP kernel similarity for circRNAs, the disease GIP kernel similarity was measured based on disease–miRNA interaction profiles. It is defined as:

(7) $$\begin{array}{c} SD\_GIP\left(d\left(i\right),d\left(j\right)\right)=exp\left(-\gamma_{d}{∥d\left(i\right)-d\left(j\right)∥}^{2}\right)\\ \gamma_{d}=\frac{1}{\frac{1}{n_{d}}\sum_{i=1}^{n_{d}}{∥d\left(i\right)∥}^{2}} \end{array}$$

where the disease interaction profiles are represented by *d*(*i*), a binary vector that encodes the interaction between disease *i* and each miRNA, i.e., the *i*-th row of association matrix *O*. The parameter *γ_*d*_* is also used to control the kernel bandwidth, and *n*_*d*_ is the number of diseases.

#### Other Disease Similarities

Besides disease symptom similarity, disease sematic similarity, and GIP kernel similarity, disease similarities can also be measured using the Lin (1998), PSB (Mathur and Dinakarpandian, 2012), Resnik (1995), and SemFunSim (Cheng et al., 2014) methods based on the DincRNA database (Cheng et al., 2018). Four disease similarity kernels were constructed using these methods and denoted *SD_Lin*, *SD_PSB*, *SD_Resnik*, and *SD_SemFunSim*, respectively.

### Similarity Kernel Fusion

Next, we used the similarity kernel fusion method to integrate four circRNA similarity kernels and seven disease similarity kernels (Jiang et al., 2018). Let *S*_*c,m*_ (*m* = 1,2,…4) represent the four circRNA similarity kernels and *S*_*d,n*_ (*n* = 1,2,…7) the seven disease similarity kernels, respectively.

First, each original similarity kernel for circRNAs was normalized using Eq. (8):

(8) $$NS_{c,m}\left(c_{i},c_{j}\right)=\frac{S_{c,m}\left(c_{i},c_{j}\right)}{\sum_{c_{k}\in C}S_{c,m}\left(c_{k},c_{j}\right)}$$

where *NS*_*c,m*_ denotes a normalized similarity kernel for circRNAs that satisfies ∑_*c*_*k*_ ∈ *C*_*NS*_*c*,*m*_(*c*_*k*_,*c*_*j*_) = 1.

Then, a sparse kernel for each circRNA similarity kernel was constructed using Eq. (9):

(9) $$F_{c,m}\left(c_{i},c_{j}\right)=\left\{ \begin{array}{cc} \frac{S_{c,m}\left(c_{i},c_{j}\right)}{\sum_{c_{k}\in N_{i}}S_{c,m}\left(c_{i},c_{k}\right)}\; & c_{j}\in N_{i}\\ 0\; & c_{j}\notin N_{i} \end{array}\right.$$

where *F*_*c,m*_ is a sparse kernel satisfying ∑_*c*_*j*_ ∈ *C*_*F*_*c*,*m*_(*c*_*k*_,*c*_*j*_) = 1, and *N*_*i*_ is a set of *c*_*i*_’s neighbors including *c*_*i*_ itself.

The four circRNA similarity kernels were computed using Eq. (10):

(10) $$\begin{array}{c} SC_{c,m}^{t+1}=\alpha \left(F_{c,m}\times \frac{\sum_{r\neq 1}SC_{c,r}^{t}}{2}\times F_{c,m}^{T}\right)\\ \;+\left(1-\alpha \right)\left(\frac{\sum_{r\neq 1}SC_{c,r}^{0}}{2}\right)\;\alpha \in \left(0,1\right) \end{array}$$

where $SC_{c,m}^{t+1}$ is the status matrix of *m*-th circRNA similarity kernel after *t*+1 iterations, and $SC_{c,r}^{0}$ denotes the initial status of *SC*_*c,r*_.

After *t*+1 steps, the overall kernel for circRNAs was calculated using Eq. (11):

(11) $$S_{c}=\frac{1}{4}\sum_{m=1}^{4}SC_{c,m}^{t+1}$$

Furthermore, a weight matrix *w*_*c*_ was used to eliminate the noise in matrix *S*_*c*_, and the fused circRNA similarity kernel was computed using Eq. (12):

(12) $$S_{c}^{*}=w_{c}\circ S_{c}$$

(13) $$w_{c}(c_{i},c_{j})=\left\{ \begin{array}{lc} 1 & \mathrm{if}\;c_{i}\in N_{j}\;\mathrm{and}\;c_{j}\in N_{i}\\ 0 & \mathrm{if}\;c_{i}\notin N_{j}\;\mathrm{and}\;c_{j}\notin N_{i}\\ 0.5 & \mathrm{otherwise} \end{array}\right.$$

Similarly, the seven disease similarity kernels were fused to form one disease similarity kernel, denoted by $S_{d}^{*}$.

### Construction of the Feature Matrix

**FIGURE 2:**
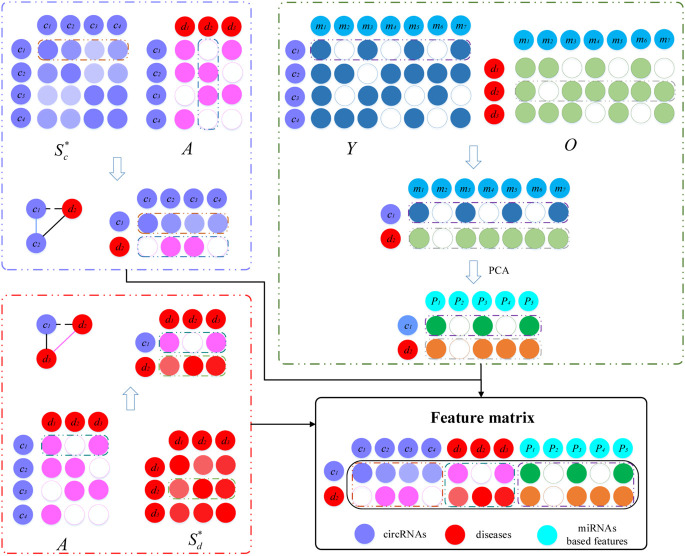
Establishment of the feature matrix of circRNA *c*_1_ and disease *d*_2_. Based on three premises, *c*_1_-*d*_2_ feature matrix is constructed by combing fused similarities and associations among circRNAs, diseases and miRNAs.

The feature matrix for each circRNA–disease pair was constructed by incorporating the fused circRNA similarity, fused disease similarity, circRNA–miRNA interactions, circRNA–disease associations, and disease–miRNA associations (Figure 2). In the construction process of the feature matrix, three biological premises were used. Here, we take the construction of the *c*_1_-*d*_2_ feature matrix as an example. Based on the premise that the circRNAs should be more similar that have interaction with circRNA similarities and circRNA–disease associations, the first part of the feature matrix consists of the similarity between *c*_1_ and all circRNAs, and the associations of *d*_2_ with all circRNAs. If circRNA *c*_1_ and *c*_2_ or other circRNAs have similar functions, and at the same time *d*_2_ has been shown to be associated with these circRNAs, *c*_1_ has a large probability associated with *d*_2_. The dimension of the first part of the feature matrix is 2 × *n*_*c*_. Similarly, based on the premise that diseases should be more similar that have interaction with disease similarities and circRNA–disease associations, we integrate the associations between circRNA *c*_1_ and all diseases, as well as the similarities between disease *d*_2_ and all diseases. The second part of the feature matrix has dimension 2 × *n*_*d*_. In addition, circRNA–miRNA and miRNA–disease is integrated to capture the relation features. When *c*_1_ and *d*_2_ have interactions with common miRNAs, they are more likely to be associated with each other. The interactions between *c*_1_ and various miRNAs, as well as the associations between *d*_2_ and miRNAs, are integrated to construct a matrix with dimension 2 × *n*_*m*_. However, the matrix is very sparse, so we perform principal component analysis (PCA) to obtain miRNA-based features for the *c*_1_-*d*_2_ pair with dimension 2 × *n*_*p*_ (*n*_*p*_ is set as 50). Finally, we concatenate these three matrices to form the feature matrix of circRNA *c*_1_ and disease *d*_2_ with dimension 2 × (*n*_*c*_+*n*_*d*_+*n*_*p*_).

### Identification of CircRNA–Disease Associations Based on CNN

**FIGURE 3:**
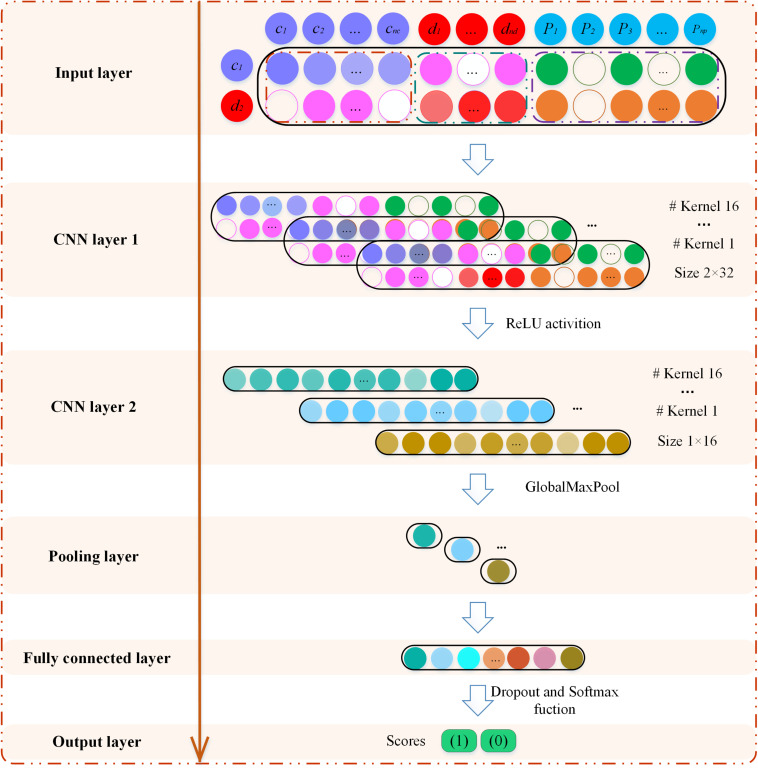
Graphical illustration of the MSFCNN architecture. The feature matrix of circRNA *c*_1_ and disease *d*_2_ is input to the convolution neural network model to learn global deep representation between *c*_1_ and *d*_2_.

The MSFCNN architecture consists of an input layer, two convolutions, and an activation layer, polling layer, fully connected layer, and softmax layer (Figure 3). The feature matrix *X* of node pairs is used as an input to the CNN architecture to learn the representations of node-pair circRNAs and diseases. The MSFCNN can be summarized as:

(14) $$Out=f^{Softmax}f^{Fully\_connected}f^{GlobalMaxPool}f^{Conv2D\_ReLU}f^{Conv2D\_ReLU}\left(X\right)$$

where *X* is the feature matrix that is fed to the two-dimensional convolution (Conv2D) layer. In the first convolutional layer, if the number of filters is *n*_*conv1*_, the width of the kernel is *n*_*w*_, and its length is set as *n*_*l*_. The convolution filters are indicated as *W*_*conv1*_∈*R^*nconv1* × *nw* × *nl*^*, and the feature maps are Z_*conv1*_∈*R^ nconv1 × (2–nw+1) × (nc+*nd*+*np–nl*+1)^*. The convolution process can be described as follows:

(15) $$X_{k,i,j}=X\left(i:i+n_{w},j:j+n_{l}\right)\;X_{k,i,j}\in R^{n_{w}\times n_{l}}$$

(16) $$\begin{array}{c} Z_{conv1,k}\left(i,j\right)=g\left(W_{conv1}\left(k,:,:\right)*X_{conv1,i,j}+b_{conv1}\left(k\right)\right)\\ k\in \left[1,n_{conv1}\right],i\in \left[1,2\right],j\in \left[1,n_{c}+n_{d}+n_{p}-n_{l}+1\right] \end{array},$$

where *X*(*i*,*j*) is the element of matrix *X* in the *i*-th row and *j*-th column, and *X*_*k,i,j*_ represents the region in the filter where the *k*-th filter slides to the position *X*(*i*,*j*). *g* is a rectified linear units (*relu*) function (Nair and Hinton, 2010), *b*_*conv1*_ is the bias vector, ^∗^ represents the convolution operation, and *Z*_*conv1,k*_(*i*,*j*) represents the convolution result of the *k*-th filter sliding to the *j*-th column of the *i*-th row.

Similarly, the second Conv2D layer is also used to learn the higher-level features. To compress data and reduce over-fitting, the polling layer is used to obtain robust features. Here, the max-pooling operation is employed for each feature map (Collobert et al., 2011). Then, the outputs of the pooling layer are concatenated together from all kernels into one feature vector and input into the fully connected layer. The nonlinear softmax activation function is used to perform the task of classification.

To avoid over-fitting, a dropout layer is implemented before the output, in which the output of every neuron is set to zero with a probability of 0.5. The dropped-out neurons are not included in the forward pass or the back-propagation (Hinton et al., 2012).

### Prediction of Novel CircRNA–Disease Associations

Next, we used all the positive and negative circRNA–disease association samples to train the MSFCNN architecture. Then, MSFCNN was used to score the unlabeled associations between circRNAs and diseases. Owing to the different negative samples used to train the model in each iteration of the five-fold cross validation (five-fold CV), we scored the candidate associations 10 times. Finally, we calculated the average scores for the candidate associations, and the candidate circRNAs related to specific diseases were analyzed using case studies.

## Results

### Performance Evaluation

The performance of MSFCNN and other conventional machine learning-based methods for predicting circRNA–disease associations was evaluated using five-fold CV. If the circRNA *c*(*i*) was found to be related to disease *d*(*j*), the node pair *c*_*i*_-*d*_*j*_ was considered as a positive example. Hence, the validated circRNA–disease associations were regarded as the positive set. However, because of the unavailability of a dataset for negative samples, we randomly selected a negative set from unobserved associations that was the same size as the positive set. All the positive samples were divided into five subsets of equal size, and each subset was tested once. For each CV, we took four positive subsets and the same number of negative subsets from five subsets to train the models; the remaining one positive subset and one negative subset were used for testing to evaluate the prediction performance. To lessen the bias resulting from sample division, we performed 10 repetitions of five-fold CV and obtained the average values of five experiments.

Receiver operating characteristic (ROC) curves were plotted to show the prediction performance by calculating the true positive rate and false positive rate. The area under the curve (AUC) was calculated to evaluate the overall performance. In addition, five metrics, precision (*Pre*), sensitivity (*Sen*), accuracy (*Acc*), *F1-score*, and Matthews’s correlation coefficient (*MCC*) were used to evaluate the capability of the MSFCNN model. The detailed calculation of these metrics was as follows:

(17) $$Pre=\frac{TP}{TP+FP}$$

(18) $$Sen=\frac{TP}{TP+FN}$$

(19) $$Acc=\frac{TP+TN}{TP+TN+FP+FN}$$

(20) $$F1-score=\frac{2\times Sen\times Pre}{Sen+Pre}$$

(21) $$MCC=\frac{TP*TN-FP*FN}{\sqrt{\left(TP+FN\right)*\left(TP+FP\right)*\left(TN+FN\right)*\left(TN+FP\right)}}$$

where *TP* and *TN* represent the number of true positives and true negatives, respectively, and *FP* and *FN* represent the number of positives and negatives, respectively, that were wrongly predicted.

### Parameter Setting

**FIGURE 4:**
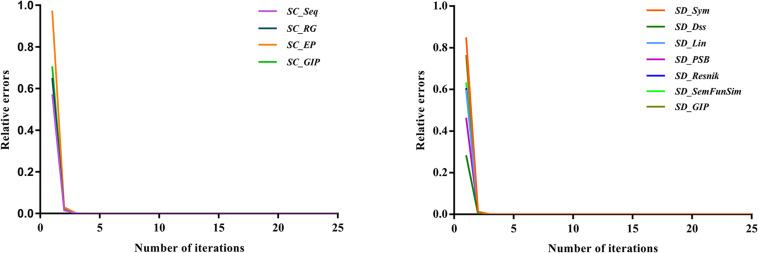
Relative errors of the SKF method with various numbers of iteration for the four circRNA similarity matrices and seven disease similarity matrices.

Convergence and parameter selection are important factors in the SKF method, that is, the number of iterations and two parameters, *α* and the size of neighbors. Following a previous study (Jiang et al., 2018), we set these two parameters to 0.1 and 36, respectively. As the number of iterations is important for the convergence of the SKF method, we also analyzed whether the number of iterations was sufficient for convergence in the four circRNA similarity kernels and seven disease similarity kernels. The relative error of the process of iteration was denoted *EC*_*t*_ and *ED*_*t*_ for circRNA similarity fusion and disease similarity fusion, respectively. The number of iterations ranged from 1 to 25 with steps of 1, and *EC*_*t*_ and *ED*_*t*_ were computed after every iteration. The convergence processes of the four circRNA similarity kernels and seven disease similarity kernels are shown in Figure 4. The results indicate that the convergence process was fast, and the *EC*_*t*_ and *ED*_*t*_ values reached 10^–10^ after 10 iterations. Therefore, we set the number of iterations to 10 for both circRNA similarity fusion and disease similarity fusion.

(22) $$EC_{t}=\frac{∥SC_{c,m}^{t+1}-SC_{c,m}^{t}∥}{∥SC_{c,m}^{t}∥}$$

(23) $$ED_{t}=\frac{∥SD_{d,n}^{t+1}-SD_{d,n}^{t}∥}{∥SD_{d,n}^{t}∥}$$

In the convolution operation of the MSFCNN model, the number of filters was set to 8. The kernel size was set to 2 × 32 in the first convolutional layer and 1 × 16 in the second convolutional layer. We implemented the MSFCNN model using the Keras 2.2.4 library in Python 3.7.3.

### Evaluation of Prediction Performance

**FIGURE 5:**
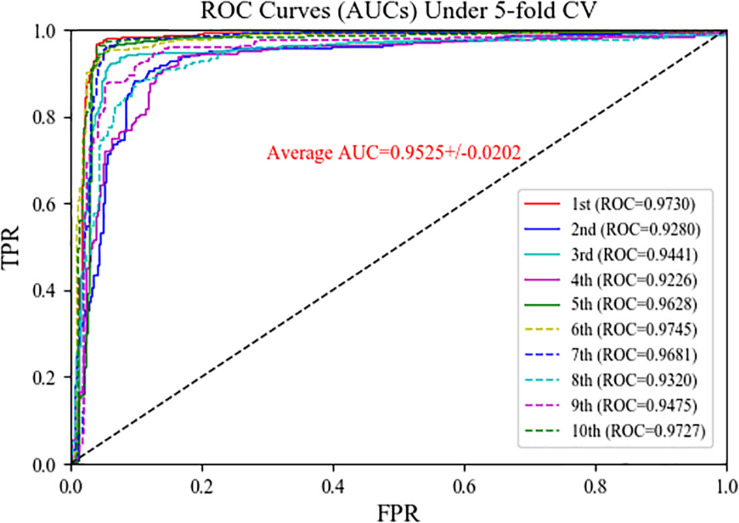
ROC curves of MSFCNN model for the task of circRNA–disease association prediction.

To assess the performance of the MSFCNN model for prediction of circRNA–disease associations, we used five-fold CV with 10 experiments (see Table 1 and Figure 5 for details). MSFCNN achieved average precision, sensitivity, *F1-score*, *Acc*, *MCC*, and AUC values of 0.9030, 0.9464, 0.9240, 0.9220, 0.8452, and 0.9525, with standard deviations of 0.0360, 0.0256, 0.0292, 0.0305, 0.0605, and 0.0202, respectively. Furthermore, the ROC curves for the MSFCNN model were at the upper left of the picture. These results indicate that our proposed model performs well in prediction of circRNA–disease associations.

**TABLE 1:** Evaluation metrics for performance of the MSFCNN approach.

Times	*Pre*	*Sen*	*F1-score*	*Acc*	*MCC*
1	0.9573	0.9677	0.9625	0.9623	0.9246
2	0.8488	0.9380	0.8912	0.8854	0.7752
3	0.9251	0.9326	0.9289	0.9286	0.8572
4	0.8660	0.9057	0.8854	0.8827	0.7663
5	0.9203	0.9650	0.9421	0.9407	0.8824
6	0.9010	0.9568	0.9281	0.9259	0.8534
7	0.9258	0.9757	0.9501	0.9488	0.8989
8	0.8641	0.9084	0.8857	0.8827	0.7665
9	0.8835	0.9407	0.9112	0.9084	0.8184
10	0.9377	0.9730	0.9550	0.9542	0.9090
Average	0.9030+/−0.0360	0.9464+/−0.0256	0.9240+/−0.0292	0.9220+/−0.0305	0.8452+/−0.0605

### Comparison With Average Kernel Fusion Strategy

**FIGURE 6:**
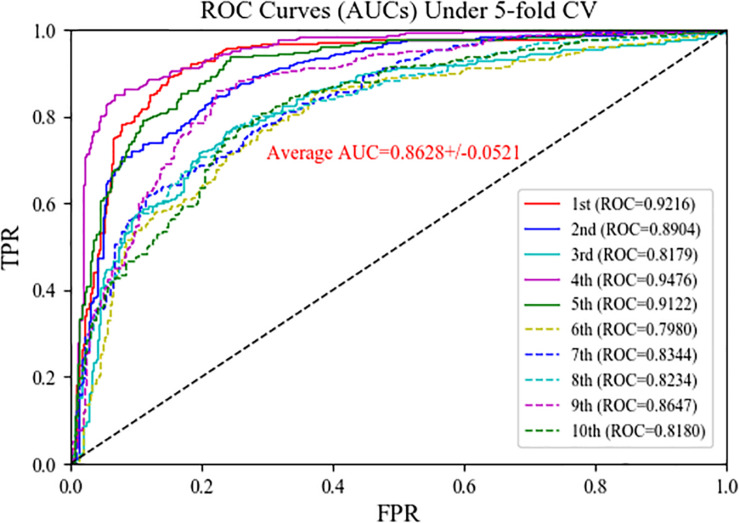
ROC curves of the MSFCNN model with average kernel fusion strategy.

In the MSFCNN model, the SKF method is used to fuse the four circRNA similarity kernels and seven disease similarity kernels into one circRNA similarity kernel and one disease similarity kernel, respectively. We compared the performance of the SKF method when integrating several similarity kernels with that of an average kernel fusion strategy. The average fusion strategy calculated the average similarity scores for four circRNA similarity matrix or seven disease similarity matrices, respectively. Five-fold CV was performed 10 times for predicting circRNA–disease associations. The average kernel fusion-based MSFCNN model had an average AUC of 0.8628 (Figure 6); by comparison, the SKF-based MSFCNN model had an AUC of 0.9525 (an improvement of 0.0897). Other evaluation metrics also indicated that the SKF method performs better than the average kernel fusion strategy in MSFCNN (Table 2). Hence, the SKF method is an effective fusion strategy for prediction of circRNA–disease associations.

**TABLE 2:** Evaluation metrics for performance of the MSFCNN model with average kernel fusion strategy.

Times	*Pre*	*Sen*	*F1-score*	*Acc*	*MCC*
1	0.8448	0.8948	0.8691	0.8653	0.7317
2	0.7889	0.8464	0.8166	0.8100	0.6216
3	0.7834	0.7116	0.7458	0.7574	0.5170
4	0.8832	0.8760	0.8796	0.8801	0.7601
5	0.8342	0.8410	0.8376	0.8369	0.6738
6	0.7186	0.7709	0.7438	0.7345	0.4702
7	0.7171	0.7925	0.7529	0.7398	0.4825
8	0.7649	0.7278	0.7459	0.7520	0.5046
9	0.7778	0.8679	0.8204	0.8100	0.6242
10	0.7357	0.7951	0.7642	0.7547	0.5111
Average	0.7848+/ −0.0553	0.8123+/ −0.0629	0.7976+/ −0.0534	0.7941+/ −0.0537	0.5897+/ −0.1070

### Comparison With Conventional Machine Learning Approaches

**FIGURE 7:**
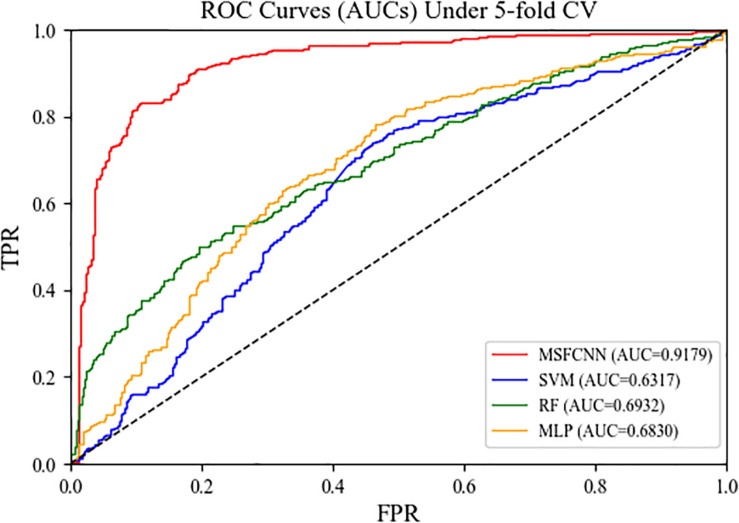
ROC curves of the MSFCNN model and other machine learning methods.

To demonstrate the reliability and robustness of the MSFCNN method, we made comparisons with state-of-the-art machine learning approaches: support vector machine (SVM), random forest (RF), and multilayer perception (MLP). For each of these machine learning approach, the feature matrix fed into the model was consistent with that used for MSFCNN to ensure the fairness of the experiments. As shown in Figure 7, the average AUC of the MSFCNN model in the five-fold CV was 0.9179 higher than those of the SVM, RF, and MLP methods. In addition, MSFCNN achieved higher precision, sensitivity, *F1-score*, *Acc*, and *MCC* values than the other machine learning approaches (Table 3). Therefore, the proposed method is more suitable than these conventional approaches for the task of circRNA–disease association prediction.

**TABLE 3:** Evaluation metrics for performance of the MSFCNN and other tmachine learning methods.

Methods	*Pre*	*Sen*	*F1-score*	*Acc*	*MCC*
MSFCNN	0.8468	0.8491	0.8479	0.8477	0.6954
SVM	0.6166	0.6415	0.6288	0.6213	0.2428
RF	0.6851	0.5337	0.6000	0.6442	0.2957
MLP	0.6455	0.6577	0.6515	0.6482	0.2965

### Case Study

**FIGURE 8:**
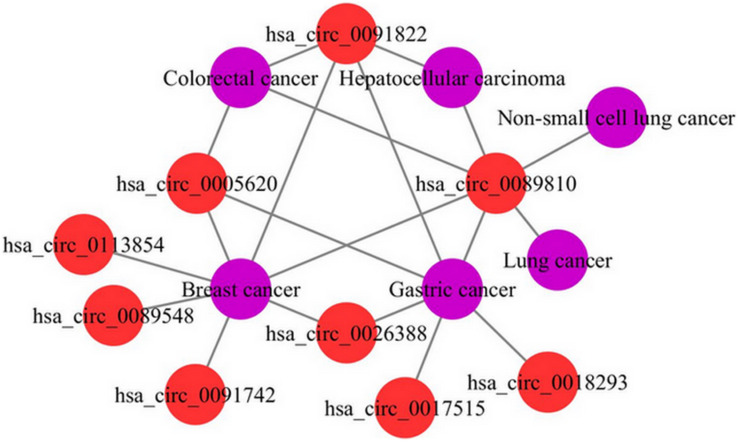
Top 20 predicted circRNA–disease associations.

To further demonstrate the ability of the MSFCNN model to discover potential circRNA–disease associations, we scored unlabeled associations between circRNAs and diseases using the trained model. Average scores were obtained from 10 applications of the MSFCNN model, and candidate circRNA–disease associations were identified based on their ranked scores. Case studies were performed for breast cancer, colorectal cancer, hepatocellular carcinoma, and acute myeloid leukemia. Some of the predicted specific disease-related circRNAs were found in the Circ2Traits database (Ghosal et al., 2013), which collects circRNAs and miRNAs related to diseases and traits (Table 4). In addition, we plotted the top 20 predicted circRNA–disease associations; the results show that these circRNAs may be related to the same diseases, and the diseases may also be associated with the same circRNAs (Figure 8). Hence, these results show that the MSFCNN model could be an effective tool for the prediction of circRNA–disease associations.

**TABLE 4:** Candidate circRNAs predicted by the MSFCNN model for four diseases.

Diseases	circRNAs	Rank	Evidence
Acute myeloid leukemia	hsa_circ_0000677	3	Circ2Traits
	hsa_circ_0000175	6	Circ2Traits
Breast cancer	hsa_circ_0000677	8	Circ2Traits
	hsa_circ_0000175	11	Circ2Traits
	hsa_circ_0001417	25	Circ2Traits
Colorectal cancer	hsa_circ_0001417	16	Circ2Traits
	hsa_circ_0000175	19	Circ2Traits
	hsa_circ_0001283	40	Circ2Traits
	hsa_circ_0000615	56	Circ2Traits
Hepatocellular	hsa_circ_0000677	10	Circ2Traits
	hsa_circ_0001417	24	Circ2Traits
	hsa_circ_0001283	48	Circ2Traits

## Conclusion

Prioritizing potential disease-related circRNAs based on various types of prior information is beneficial to understanding disease mechanisms, diagnosis, and treatment. In this study, we developed a novel computational method named MSFCNN to predict potential circRNA–disease associations, using a two-layer two-dimensional CNN and integrating multiple biological data. First, one of the crucial technical points for predicting circRNA–disease associations is the similarity calculation for circRNA–circRNA and disease–disease pairs. Therefore, we calculated four circRNA similarity kernels and seven disease similarity kernels based on multiple biological and topological information. In addition, similarity kernel fusion was used to integrate various similarity kernels into one circRNA similarity kernel and one disease similarity kernel. Based on these fused similarity kernels and interactions/features among circRNAs, miRNA, and diseases, a feature matrix was constructed for each circRNA–disease pair. Finally, a two-layer CNN architecture was used to predict circRNA–disease associations. The MSFCNN approach showed good performance based on the five-fold CV, outperforming the SVM, RF, and MLP classifiers. Furthermore, case studies of breast cancer, colorectal cancer, hepatocellular carcinoma, and acute myeloid leukemia demonstrated that the MSFCNN framework could be an effective tool for successfully inferring potential circRNA–disease associations and providing a basis for biological validation.

The good performance of MSFCNN method mainly conclude following aspects. Firstly, multiple similarity kernels for circRNAs and diseases are effectively introduced to measure the biological and topological features of circRNAs and diseases. Secondly, the relationships of circRNA–miRNA and disease–miRNA are also used to construct the feature matrix for each circRNA–disease pair. Furthermore, the application of CNN architecture guarantees the effectiveness of learning the meaning of combinations of features from the feature matrix. Hence, MSFCNN method is an effective biomedical resource to predict the circRNA–disease associations.

Despite its promising prediction performance, the MSFCNN approach has some limitations. First, incomplete and noisy circRNA–disease associations were used as positive samples, and negative samples are randomly selected, limiting the prediction performance. This could be improved as more associations are discovered. Furthermore, more reliable biological information should be considered, such as circRNA coding potential and circRNA functional information, as well as disease phenotypes and functional information, etc. In addition, optional similarity measurements would be integrated based on comparing the prediction results of different similarity measures. Therefore, more data sources should be collected, and a more effective model needs to be developed to address the above limitations.

## Data Availability Statement

Publicly available datasets were analyzed in this study. This data can be found here: http://bioinfo.snnu.edu.cn/CircR2Disease/, http://www.circbank.cn/, https://disease-ontology.org/, http://bioannotation.cn:18080/DincRNAClient/#/Home, https://www.nlm.nih.gov/mesh/, http://www.cuilab.cn/hmdd, and http://www.exorbase.org.

## Author Contributions

XL and YP conceptualized the study. CF and XL performed the data collection, designed the method, and drafted the manuscript. All authors read and approved the final version of the manuscript.

## Conflict of Interest

The authors declare that the research was conducted in the absence of any commercial or financial relationships that could be construed as a potential conflict of interest.
